# C6: A Monoclonal Antibody Specific for a Fibronectin Epitope Situated at the Interface between the Oncofoetal Extra-Domain B and the Repeat III8

**DOI:** 10.1371/journal.pone.0148103

**Published:** 2016-02-11

**Authors:** Elisa Ventura, Cinzia Cordazzo, Rodolfo Quarto, Luciano Zardi, Camillo Rosano

**Affiliations:** 1 Laboratory of Oncology, G. Gaslini Institute, Genova, Italy; 2 Sirius-biotech, c/o Advanced Biotechnology Center, Genova, Italy; 3 Laboratory of Stem Cells, University of Genoa and Laboratory of Regenerative Medicine, IRCCS AOU San Martino-IST, Genova, Italy; 4 Laboratory of Biopolymers and Proteomics, IRCCS AOU San Martino-IST, Genova, Italy; University of North Carolina at Chapel Hill, UNITED STATES

## Abstract

**Background:**

Fibronectin (FN) is a large multidomain molecule that is involved in many cellular processes. Different FN isoforms arise from alternative splicing of the pre-mRNA including, most notably, the FN isoform that contains the “extra-domain-B” (ED-B). The FN isoform containing ED-B (known as B-FN) is undetectable in healthy adult tissues but is present in large amounts in neoplastic and foetal tissues as well as on the blood vessels during angiogenesis. Thus, antibodies specific for B-FN can be useful for detecting and targeting neoplastic tissues *in vivo*. We previously characterised C6, a new monoclonal antibody specific for human B-FN and we suggested that it reacts with the B-C loop of the type III repeat 8 which is masked in FN isoforms lacking ED-B and that the insertion of ED-B in FN molecules unmasked it. Here we have now consolidated and refined the characterization of this B-FN specific antibody demonstrating that the epitope recognized by C6 also includes loop E-F of ED-B.

**Methodology:**

We built the three dimensional model of the variable regions of the mAb C6 and of the FN fragment EDB-III8 and performed protein:protein docking simulation using the web server ClusPro2.0. To confirm the data obtained by protein:protein docking we generated mutant fragments of the recombinant FN fragment EDB-III8 and tested their reactivity with C6.

**Conclusion:**

The monoclonal antibody C6 reacts with an epitope formed by the B-C loop of domain III8 and the E-F loop of ED-B. Both loops are required for an immunological reaction, thus this monoclonal is strictly specific for B-FN but the part of the epitope on III8 confers the human specificity.

## Introduction

Fibronectin (FN) is a multi-domain molecule present in the extracellular matrix (ECM) and in body fluids. It is a dimer of two subunits of about 220/250kDa, linked at the C-termini by two disulfide bonds and each monomer consists of three types of repeating units. FN is involved in many cellular processes and different FN isoforms arise from the alternative splicing of its pre-mRNA [1–2]. In particular, the FN isoform containing the extra-domain B (ED-B), a complete FN type III repeat formed by 91 amino acids, is expressed only during physiological or pathological tissue remodelling such as in embryogenesis, wound healing, in uterus and ovary during the female reproductive cycle, in tumorigenesis and in degenerative chronic inflammatory diseases. The ED-B primary structure is highly conserved in different species, having 100% homology in all mammals thus far tested and 96% homology with a similar domain in chicken.

The FN isoform containing ED-B (B-FN) is undetectable in healthy adult tissues but its expression levels are highly increased in tumour tissues and it accumulates around neovasculature during angiogenesis. This makes it one of the oncologist’s best markers of angiogenesis and neoplastic tissues [3–8]. The demonstration that monoclonal antibodies to B-FN can be used to selectively deliver therapeutic substances to diseased tissues [9] prompted the generation of human recombinant antibodies for preclinical and clinical diagnostic and therapeutic purposes [10–13].The biological function(s) of B-FN are still unclear, however it has been suggested that B-FN increases vascular endothelial growth factor (VEGF) expression, endothelial proliferation and tube formation [14]. More recently Kraft et al [15] reported that B-FN enhances phagocytosis more than plasma FN and that this enhancements is mediated by the integrin alphaVbeta3. On the whole the biological activities are mediated by exposed loops located mainly at the inter-domain interface, therefore the insertion of ED-B within repeat III7 and III8 modifies the domain-domain interface and would be expected to lead to changes in biological activities [16].

We have previously described C6, a monoclonal antibody specific for human B-FN [17]. Using various recombinant FN fragments containing mutations we concluded that its epitope was located within the loop B-C of III8 and we speculated that, in FN isoforms lacking ED-B, this loop is masked [18]. Here, to better understand the interaction between human B-FN and C6, we performed protein:protein docking simulation of the three dimensional models of the scFv of the mAb C6 and of the FN recombinant fragment containing the type III domains B and 8. The results confirm the interaction with the loop B-C of domain III8 but also with the loop E-F of ED-B. Further experiments using a FN fragment with mutation on ED-B confirmed that its loop E-F is part of the epitope recognized by C6.

## Results and Discussion

**Fig 1:**
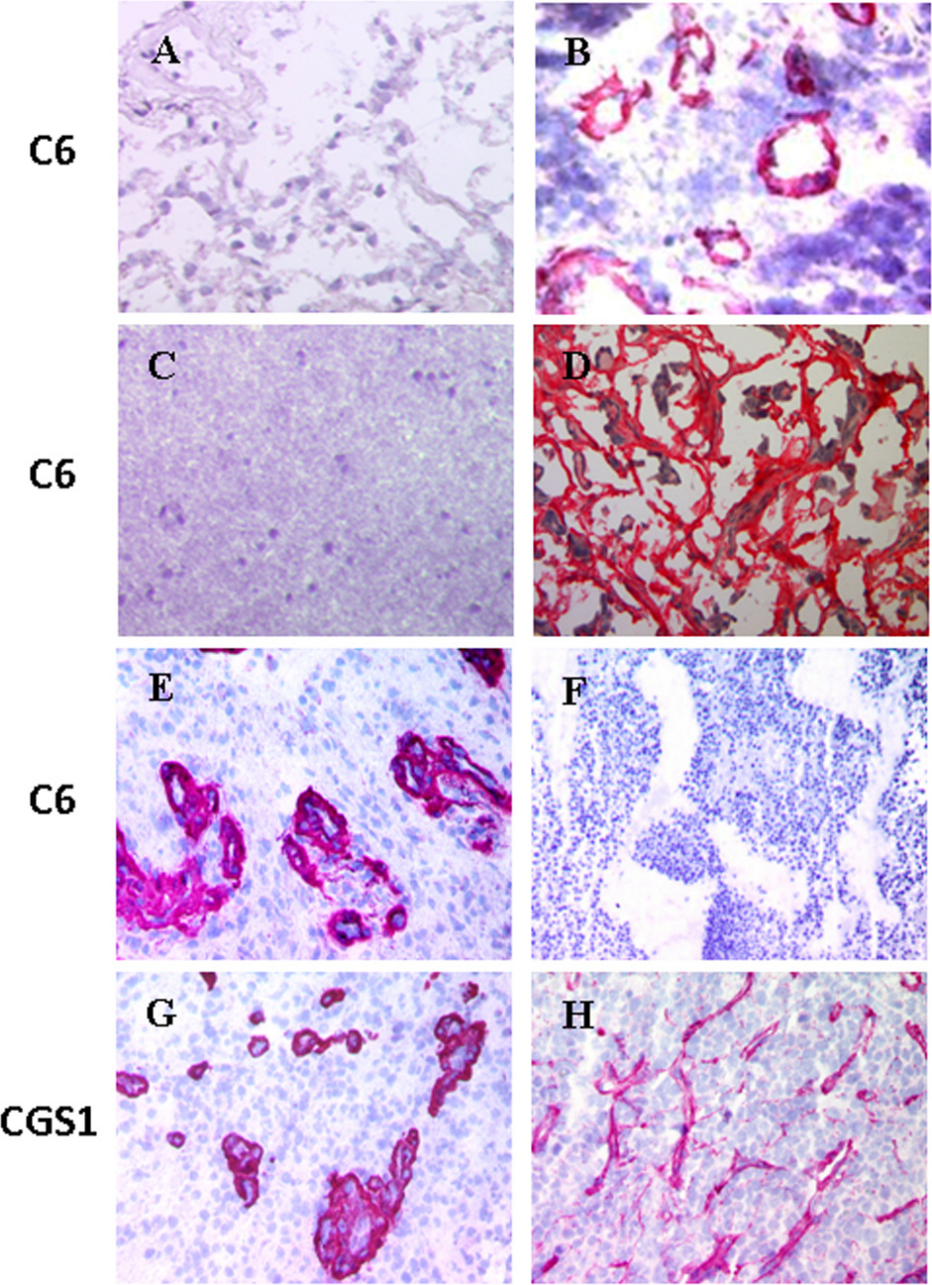
Immunohistochemistry experiments using the mAb C6 on cryostat sections. A) Normal human lung; B) human lung adenocarcinoma; C) Normal human brain; D) Human mesothelioma; E) Human glioblastoma; F) Murine teratocarcinoma. Immunohistochemistry experiments using the recombinant antibody CGS1 on cryostat sections of human glioblastoma (G) and murine teratocarcinoma (H). CGS1 reacts directly with the ED-B (10) and thus, contrary of C6, it also reacts with murine tumours. C6 reacts, in an identical manner of CGS1, with normal and cancer human tissues but not with murine tumours (Modified from ref.[17] under CC-BY license, with permission f from John Wiley and Sons, original copyright 2009.).

In immunohistochemistry experiments on human tissue, the mAb C6 behaves exactly as an antibody that reacts directly with the ED-B, as it shows no reaction with healthy adult human tissues but shows a strong reaction with cancer tissue. However, unlike other ED-B specific antibodies, C6 shows no reaction with mouse tumours (Fig 1 and [17]). The absence of reaction of C6 with murine B-FN suggested that it did not react simply with ED-B, because human and mouse ED-B have a homology of 100 percent and other ED-B antibodies react equally well with human and mouse B-FN. In fact, the mAb C6 specifically recognises human B-FN and human FN recombinant fragments containing at least the type III domains B and 8. C6 does not react with mouse B-FN, human recombinant fragments formed by the type III domains 7–8, 7-B, isolated ED-B and it interacts only weakly with the isolated III8 [17]. Balza et al. [17] located the epitope recognized by C6 on III8 and excluded the possibility that the epitope was located on ED-B since its sequence is highly conserved, having 100% homology in all mammalians thus far tested it should react with the B-FN of all mammalian species while C6 is specific only for the human B-FN.

Furthermore Ventura et al. [18] compared the sequences of human and mouse III8, and since they only differ in four residues the generation of chimerical mutants, inserting residues present in mouse FN in the human recombinant fragment EDB-III8 allowed the localization of the epitope on the loop B-C. In fact it was sufficient to mutate the Asp1385 located within the loop B-C to a Glu, present in the same position in mouse, to completely abolish the ability of the mAb C6 to react with the FN fragment formed by the type III domains B-8 [18].

**Fig 2:**
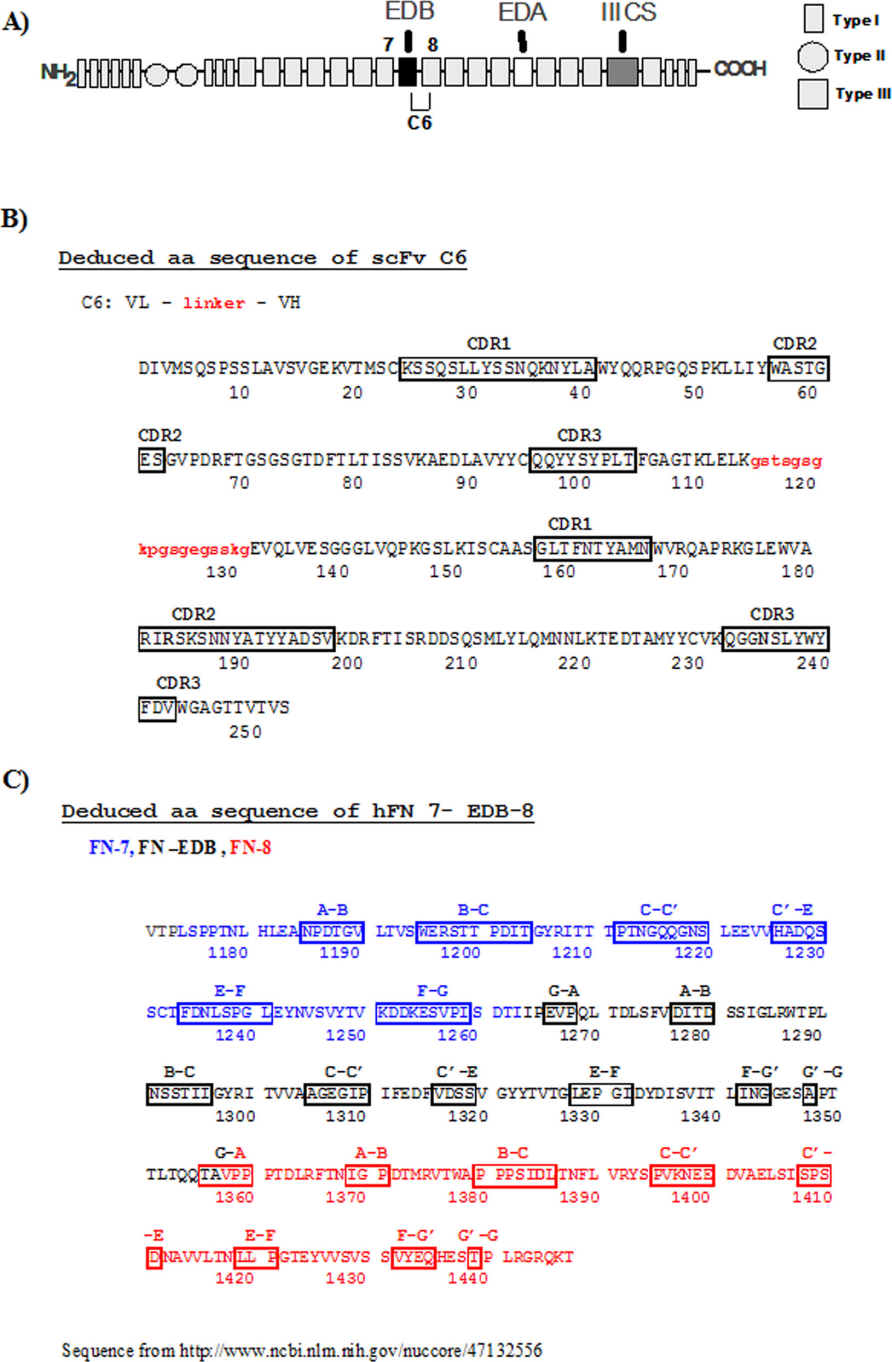
Structures of FN and of the scFv C6. **A)** Model of the domain structure of a FN subunit. The three different types of repeats, the three sites of alternative splicing (ED-A, ED-B, IIICS) and the specificity of the mAb C6 for the interface between type III repeats B and 8 are shown. **B)** Sequence of the scFv C6; the linker between the VL and the VH is in red; the CDRs are framed. Amino acids involved in the interaction with C6 are in bold. **C)** Sequence of the FN type III repeats numbers 7 (blue), B (black) and 8 (red); the various loops between the beta sheets structures are framed. The amino acids involved in the interaction with C6 are in bold. Sequence from http://www.ncbi.nlm.gov.nuccore/47132556.

Since C6 does not react with the fragment formed by type III domains 7 and 8 but reacts with the fragment B-8, Ventura et al. suggested that the epitope recognized by C6 was within the loop BC of III8, and speculated that this epitope was masked in FN molecules lacking ED-B and unmasked when ED-B was inserted within the FN molecule [18]. Fig 2 shows the domain structure of FN (A); the amino acid sequence of the scFv C6 with its complementarity determining region (CDR) (B); the amino acid sequence of the recombinant fragment containing the domains of type III 7-B-8 and the loops of the various repeats (C).

Here, in order to better understand the interaction between the antibody C6 and FN we built the three dimensional model of the scFcv C6 [17] and of the FN fragment EDB-III8 and performed protein:protein docking simulation using the web server ClusPro2.0 [19]. The most probable binding mode of C6 to FN was the lowest energy solution belonging to the most populated cluster, as determined by the program. This cluster was formed by 152 individuals and it was fairly separated by the second and the third ranked ensembles, populated by 77 and 71 individuals respectively.

**Fig 3:**
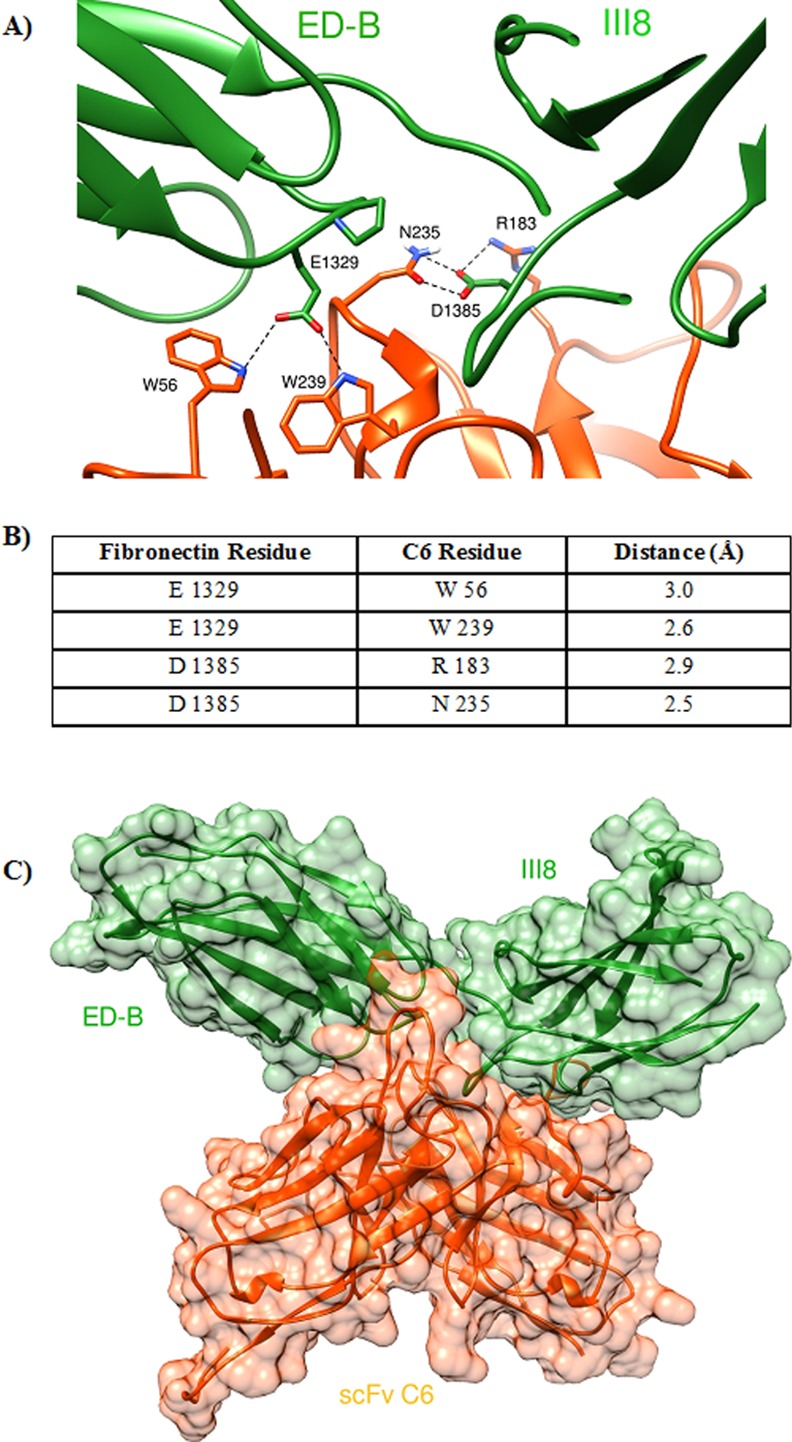
Interaction between C6 and the FN recombinant fragment formed by the type III repeats B and 8. **A)** Schematic representation of the main interactions between the two proteins. The type III domains B and 8 are drawn as green ribbons, the scFv C6 is orange. Residues involved in the binding are reported as sticks, hydrogen bonds between amino acids are shown as dotted black lines. **B)** Residues of the scFv and of the type III repeats B and 8 that interact. The distances are displayed. **C)** Representation of the interaction between the type III domains B-8 (green surface) and the scFv C6 (orange surface).

The results of docking simulation, shown in Fig 3, clearly indicated the FN D1385, located on the III8 could be a component of the epitope as previously demonstrated by Ventura et al. using chimerical mutants of FN fragments [18]. In fact the residues R183 and N235 of C6, on the VH-CDR2 and VH-CDR3 respectively, are at a distance from D385 of III8 of 2.5–3 Angstrom, which is a distance that allows hydrogen bond formation (Fig 3A and 3B). However, the results also indicated that Glu1329, located on the ED-B, is a possible component of the epitope. In fact the residues W56 and W239 of C6, located on VL-CDR2 and VH-CDR3 respectively also have a distance of 2.5–3 Angstrom from E1329 of ED-B (Fig 3A and 3B). Fig 3C shows a representation of the interaction between the type III domains B-8 and the scFv C6.

**Fig 4:**
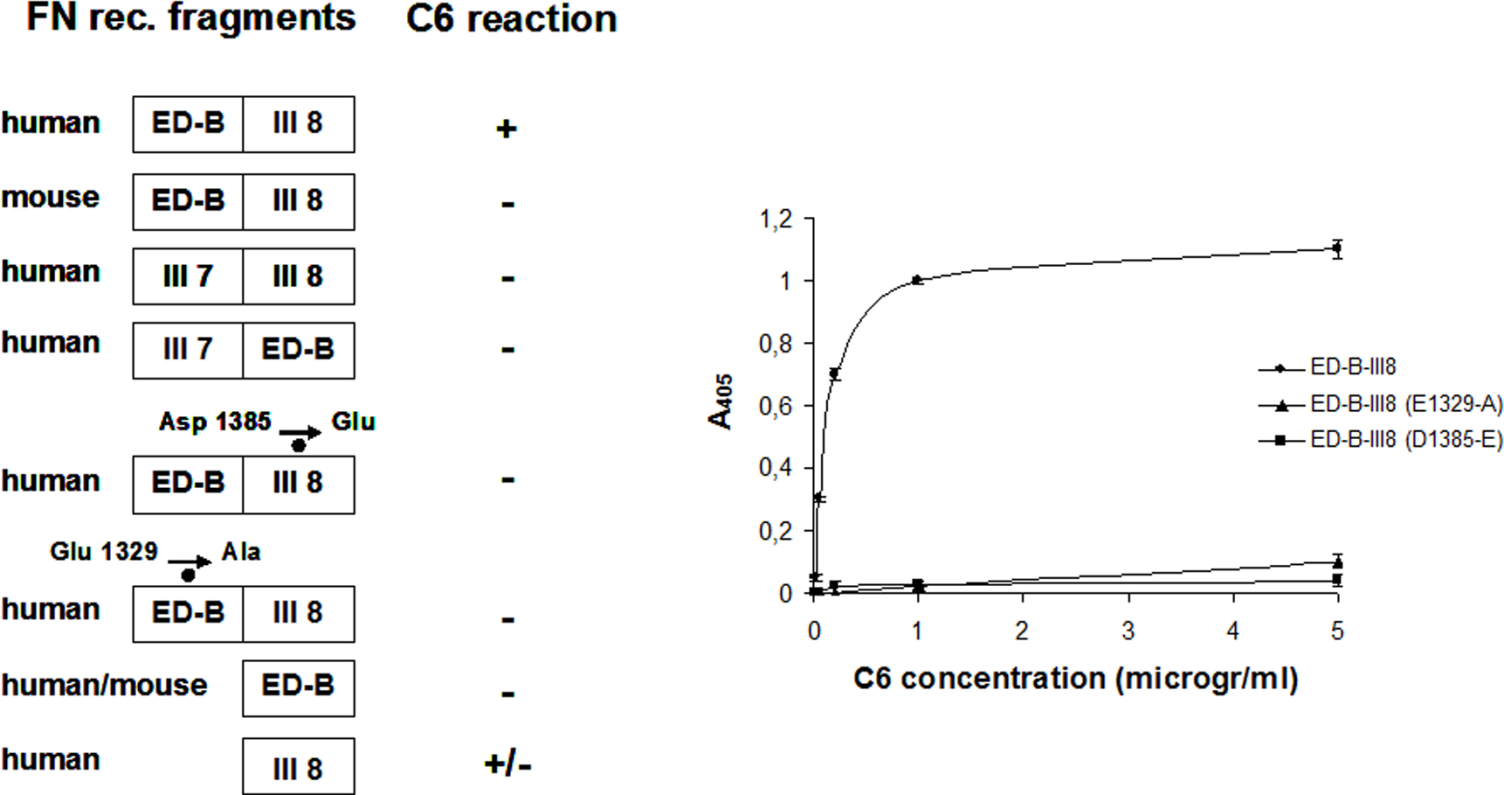
Reactivity of the mAb C6 with FN recombinant fragments. **A)** Different FN recombinant fragments tested with the mAb C6. **B)** Reactivity in ELISA of various concentrations of the mAb C6 with the FN recombinant fragment containing the type III repeats B and 8; it fragment B-8 with the mutation Glu1329Ala and fragment B-8 with the mutation Asp1385Glu.

To confirm that E1329 of the ED-B is also relevant for the C6-FN interaction, we generated a mutant of the type III repeats B-8 by substituting Glu 1329 with an Ala and tested this fragment with the antibody C6 in ELISA assay. The results shown in Fig 4 indicated that this mutant does not react with C6, thus confirming the protein:protein docking simulation that ED-B is involved in the interaction of with C6-B-FN.

Thus, the epitope of C6 encompasses both E1329 on ED-B and D1385 on III8, and the simultaneous presence of both residues is required for reaction with C6; neither of the two alone is sufficient to ensure the interaction of C6 with B-FN. The specificity of C6 for the B-FN isoform is due to the E-F loop of the ED-B whereas the human specificity is due to the B-C loop of III8, since in the majority of other mammalian species instead of the D1385 there is E, and this is sufficient to abolish the reactivity of FN with C6.

Bencharit et al. [16] reported various differences at the interface of III8 with ED-B or III7; the main differences concern the conformation and location of the loops AB, CC’ and EF of ED-B that are different from those of III7. Furthermore the inter-domain linker of ED-B and III8 buries 416Å^2^ while that between III7 and III8 buries 578Å^2^ [16]. The above data explain the absence of interaction between III7-III8 and C6.

In conclusion, here we consolidate and refine the previous report about the epitope recognized by the mAb C6; we report that its epitope encompassed the loop B-C of III8 (which confers the specificity for human B-FN) and also the loop E-F of ED-B (that confers the specificity for B-FN). In previous works the authors did not take into account the possibility that the epitope could consist of a part of ED-B and a part of III8 [17–18]. This because it is known that the ED-B sequence is not immunogenic in mice and have never been reported mouse antibodies to ED-B. This is the first report on a murine mAb that reacts with an epitope partially formed by an ED-B sequence. This peculiarity makes C6 strictly specific for human B-FN and it can therefore be used, for example, to distinguish human B-FN in models of human tumours transplanted in mice.

Considering that the mAb C6 interacts at the EDB and III8 interface (Fig 3C) to which possible biological functions have been attributed, C6 will also be helpful in discovering biological activities of B-FN. For example C6 can be tested for its the ability to inhibit the functions that have been attributed to the B-FN such as phagocytosis and its ability to increase the expression of VEGF [14–15].

There are other B-FN-specific antibodies, such as BC-1 that was generated over 25 years ago. BC-1 [4] was extensively used to demonstrate that B-FN is an excellent marker of angiogenesis and that mAbs to ED-B can be used *in vivo* to selectively target tumours. BC-1 recognizes an epitope, localized on the repeat III7, which is hindered in FN molecules lacking ED-B and exposed in FN molecules containing ED-B. The results obtained with BC-1 prompted the generation of recombinant antibodies directly reacting with ED-B [10–13]. These antibodies have been used for generation of radio- immunoconjugates as well as fusion proteins with cytokines such as TNF and IL2, for selective delivery of drugs to the tumours and they are currently used in both diagnostic and therapeutics clinical trials [10–13]. However, bio-distribution experiments in tumour-bearing mice showed that C6 has a longer residence time in tumours when compared to other antibodies currently used in clinical trials [17], probably as consequence of the higher resistance of the C6 epitope to proteolytic enzyme. This makes C6 an attractive antibody for clinical application.

## Methods

### Protein modelling and docking simulations

Three dimensional model structures of the scFv C6 was built using the Phyre2 program [19] and further minimized by simulated annealing using the program CNS [20]. Docking calculations were carried out by the web server ClusPro 2.0 [21] using the atomic X-ray structure of FN domains 7-B-8-9 as a target ([22]; PDB code 3T1W) through a systematic rigid-body search of one molecule translated and rotated about the other. ClusPro2.0 was run using the standard parameters after removing the ions and water molecules from the FN coordinate file. The intermolecular energies, for all configurations generated by this search, were calculated as the sum of electrostatic and van der Waals energies. The software chooses the lowest energy solutions, clusters them together and considers the lowest energy individual of the most populated cluster as the best candidate.

### Preparation of wild type and mutated human FN recombinant fragments and ELISA assay

The human recombinant FN fragments EDB-III8 as well as its mutant (D1385—E) were previously described [17]. The cDNA encoding for the human FN recombinant fragment EDB-III8 with the mutation (E1329—A) was obtained in two steps from the cDNA of human FNIII B-8. In the first step two fragments were amplified: 1. FN fragment corresponding to amino acids 1266–1332 with mutation (E1329—A) obtained with primer forward TI-147 (5’-ctcgaattcaagaggtgccccaactcact-3’), including the *Eco* restriction site, and primer reverse sb-18 (5’-aatgcccggcgccagccctgt-3’) allowing the substitution (E1329-A); 2. FN fragment corresponding to amino acids 1326–1447 with mutation (E1329—A) obtained with primer forward sb-17 (5’-acagggctggcgccgggcatt-3’), allowing the substitution (E1329-A) and complementary to primer sb-18, and the primer reverse sb-9 (5’-ctcgcggccgctcatcatgttttctgtcttcctct-3’) including the *Not* restriction site and two stop codons. In the second step the two cDNA fragments were PCR assembled with primers TI-147 and sb-9. The obtained cDNA fragment was digested *Eco/Not* and inserted into *Eco/Not* digested pProEX-1 (Life Technologies, Gaithersburg, MA, USA). All PCR reactions were performed with high fidelity PWO DNA Polymerase (Roche Diagnostics, Basel, Switzerland) following the manufacturer’s instruction. The restriction enzymes were from Roche Diagnostics. The DNA construct was used to transform DH5α competent bacteria cells. All FN recombinant fragments were purified from the bacterial lysate on Ni-NTA columns (Qiagen, Hilden Germany) using the His6 tag at the N-termini of the FN fragments. The purified FN fragments were analyzed by SDS-PAGE as previously described. The reactivity of monoclonal C6 with the FN fragments was assessed by ELISA assay as previously described [17]. Recombinant FN fragments containing ED-B were used in immunohistochemistry control experiments to inhibit the antibodies.
